# Planting and seasonal and circadian evaluation of a thymol-type oil from *Lippia thymoides* Mart. & Schauer

**DOI:** 10.1186/s13065-018-0484-4

**Published:** 2018-11-12

**Authors:** Sebastião G. Silva, Pablo Luis B. Figueiredo, Lidiane D. Nascimento, Wanessa A. da Costa, José Guilherme S. Maia, Eloisa Helena A. Andrade

**Affiliations:** 1 Programa de Pós-Graduação em Química, Universidade Federal do Pará, Belém, PA 66075-900 Brazil; 2 Programa de Pós-Graduação em Engenharia de Recursos Naturais da Amazônia, Universidade Federal do Pará, Belém, PA 66075-900 Brazil; 3 Coordenação de Botânica, Museu Paraense Emílio Goeldi, Belém, PA 66077-530 Brazil

**Keywords:** *Lippia thymoides*, Verbenaceae, Essential oil composition, Seasonal and circadian study, Thymol

## Abstract

**Background:**

The oil and extracts of *Lippia thymoides* have been used for various medicinal and food applications. Entrepreneurs in the Amazon have been considering the economic exploitation of this plant. The present study evaluated the influence of the seasonal and circadian rhythm on the yield and composition of the essential oil of leaves and thin branches of a *Lippia thymoides* specimen cultivated in Abaetetuba, State of Pará, Brazil. The constituents of the oils were identified by GC and GC–MS and with the application of multivariate analysis: Principal Component Analysis (PCA) and Hierarchical Cluster Analysis (HCA).

**Results:**

The predominance of oxygenated monoterpenes (70.6–91.8%) was observed in oils, followed by monoterpene hydrocarbons (1.2 to 21.6%) and sesquiterpene hydrocarbons (3.9 to 9.1%). Thymol, thymol acetate, γ-terpinene, *p*-cymene, and (*E*)-caryophyllene were the first compounds. The mean thymol content was higher in the rainy season (seasonal: 77.0%; circadian: 74.25%) than in the dry period (seasonal: 69.9%; circadian: 64.5%), and it was influenced by climatic variables: rainfall precipitation, solar radiation, temperature, and relative humidity. For the circadian study, PCA and HCA analysis were applied to the constituents of oils from rainy and dry periods. Two groups were formed. A higher thymol content characterized the group 1, followed by (Z)-hexen-3-ol, α-thujene, α-pinene, α-phellandrene and humulene epoxide II, in minor percent. A higher content of p-cymene formed the group 2, γ-terpinene, thymol acetate and (*E*)-caryophyllene, followed by myrcene, α-terpinene, 1,8-cineole, terpinen-4-ol, methylthymol, and germacrene D, in a low percentage.

**Conclusions:**

The different chemical profiles found in the oils of *L. thymoides* must be associated with the environmental conditions existing at its collection site. The knowledge of this variation in the oil composition is essential from the ecological and taxonomic point of view, regarding the management and economic use of the species.

## Background

*Lippia* L. is one of the largest genera of Verbenaceae, with nearly 100 species of herbs, shrubs and small trees distributed in the Neotropics and Africa [1]. *Lippia thymoides* Mart. & Schauer (Verbenaceae) [syn. *Lippia micromera* var*. tonsilis* Moldenke, *L. satureiaefolia* Mart. & Schauer, *L. thymoides* var. *macronulata* Moldenke, *L. thymoides* var. *tonsilis* (Moldenke) Moldenke [2], is an aromatic plant with a shrub size (1.0–2.0 m in height), endemic to the Northeast and Center-West of Brazil, with the distribution center in the states of Bahia and Minas Gerais, popularly known as “alecrim-do-mato”, “alecrim-do-campo” and “alecrim-de-cheiro-miúdo” [3, 4]. The plant was introduced in the Brazilian Amazon, where it is known as “manjerona”, particularly in the Municipality of Abaetetuba, State of Pará, Brazil. It is used in folk medicine, in baths for treatment of wounds, as antipyretic, digestive, in the treatment of bronchitis and rheumatism, for a headache and weakness, and as incense in the rituals of Umbanda and Candomblé [3, 5, 6].

The essential oil can undergo a qualitative and quantitative change in several stages of the vegetative life of the plant because its metabolic activity has a chemical interconnection with the medium in which it is inserted. Some environmental factors contribute to this, among them, the circadian regime, which refers to the time of collection of the plant throughout the day, and the seasonality, which represents the time of collection during the year. Thus, the production of the oils can suffer variation due to environmental changes such as temperature, relative humidity, precipitation, solar radiation, among others, occurring during the day or a certain seasonal period [7, 8].

There are few reports on the composition of the essential oil of *L. thymoides*. Two specimens from Olindina and Feira de Santana, Bahia State, Brazil, presented leaf oils with (*E*)-caryophyllene as the main component, followed by other sesquiterpene hydrocarbons in a lower percentage [9, 10]. The oil of another specimen, sampled in Belém, state of Pará, Brazil, presented thymol as the significant component [11]. Regarding the biological activity, the oils of *L. thymoides* with a predominance of (*E*)-caryophyllene, showed spasmolytic and antidiarrheal effects [12], besides antimicrobial activity and significant relaxing potential in pre-contracted smooth muscle [10]. Crude extracts of *L. thymoides* (leaves, flowers, and fine branches) also showed antimicrobial activity, healing, and anti-thermic action in rodents [13, 14].

The present study evaluated the circadian and seasonal variation of the essential oil of a thymol rich specimen of *Lippia thymoides*, previously submitted to a cultivation test in the city of Abaetetuba, State of Pará, Brazil, intending to its future economic exploitation.

## Experimental

### Planting of *Lippia thymoides*

The vegetative propagation of *Lippia thymoides* was initiated with the donation of a seedling, by a resident of the Municipality of Abaetetuba, State of Pará, Brazil. From this seedling, other two seedlings were prepared which, together with the initial seedling, formed the three matrices. Then, the plant was propagated with stem cuttings (30 stakes, 25 to 30 cm long) in disposable plastic cups, using black earth as a substrate, according to [15]. The experiment was maintained under these conditions for 5 weeks, with watering of the plants at the end of the day. Then, the 30 seedlings were transplanted to the field, arranged in two lines with a spacing of 50 cm between each seedling, without soil fertilization. Following the same methodology, after 3 months another 30 seedlings were produced. The planting occurred in the locality known as “Colônia Velha” (01°46′15.9″ S/48°47′02.2″ W), PA 151 Road, Municipality of Abaetetuba, State of Pará, Brazil.

### Plant material

For the seasonal study, the leaves and thin branches (aerial parts) of *L. thymoides* were collected monthly, between January and December, always on the 15th day, at 6 a.m. For the circadian study, the collections were carried out in February (rainy period) and September (dry period), at the hours of 6 a.m., 9 a.m., 12 a.m., 3 p.m., 6 p.m. and 9 p.m. Samples were collected in triplicate. The botanical identification was made by comparison with an authentic specimen of *Lippia thymoides* and samples of the plant (MG 213373) were incorporated into the Herbarium “João Murça Pires” of the Museu Paraense Emílio Goeldi, in the city of Belém, State of Pará, Brazil.

### Climate data

Climatic factors such as relative air humidity, temperature, and rainfall precipitation were obtained monthly from the website of the Instituto Nacional de Meteorologia (INMET, http://www.inmet.gov.br/portal/), of the Brazilian Government. The meteorological data were recorded by the automatic station A-201 of the city of Belém, with a range of 100 km. The plant cultivation area and sample collection are located in the municipality of Abaetetuba, about 52 km from the city of Belém, thus within the radius of action of the A-201 automatic station, which is equipped with a Vaisala system of meteorology, model MAWS 301 (Finland).

### Plant processing

The fresh plant material (leaves and fine branches) was cut, homogenized and submitted to hydrodistillation (65 g, 3 h) in a Clevenger type glass apparatus. After extraction, the oil was dried over anhydrous sodium sulfate. The determination of the residual water content of the plant material was carried out in a moisture-determining balance using infrared. The oil yield was calculated in % m/v (mL/100 g) [16].

### Analysis of oil composition

Qualitative analysis was carried out on a THERMO DSQ II GC–MS instrument, under the following conditions: DB-5 ms (30 m × 0.25 mm; 0.25 μm film thickness) fused-silica capillary column; programmed temperature: 60–240 °C (3 °C/min); injector temperature: 250 °C; carrier gas: helium, adjusted to a linear velocity of 32 cm/s (measured at 100 °C); injection type: splitless (2 μL of a 1:1000 hexane solution); split flow was adjusted to yield a 20:1 ratio; septum sweep was a constant 10 mL/min; EIMS: electron energy, 70 eV; temperature of ion source and connection parts: 200 °C. Quantitative data regarding the volatile constituents were obtained by peak-area normalization using a FOCUS GC/FID operated under GC–MS similar conditions, except for the carrier gas, which was nitrogen. The retention index was calculated for all the volatiles constituents using an n-alkane (C8–C40, Sigma–Aldrich) homologous series. Individual components were identified by comparison of both mass spectrum and GC retention data with authentic compounds which were previously analyzed with the aid of commercial libraries containing retention indices and mass spectra of volatile compounds commonly found in essential oils [17, 18].

### Statistical analysis

Statistical significance was assessed by the Tukey test (p < 0.05) and the Pearson correlation coefficients (R) were calculated to determine the relationship between the parameters analyzed (GraphPad Prism, version 5.0). The Principal Component Analysis (PCA) was applied to verify the interrelation in the composition of the oils of the leaves, collected at different times and months (software Minitab free 390 version, Minitab Inc., State College, PA, USA). The Hierarchical Grouping Analysis (HCA), considering the Euclidean distance and complete linkage, was used to verify the similarity of the samples of the oils, based on the distribution of the constituents selected in the PCA analysis.

## Results and discussion

The rational planting of *L. thymoides* can determine a better use for this species in the Amazon, with basis on the economic exploitation of the essential oil of some known chemical types. The crop, established in underutilized areas of secondary forests and savannas, can lead to it densification and consequent commercial exploitation.

### Planting of *L. thymoides*

A cultivation test was carried out in a dystrophic yellow latosol, medium texture, with solar radiation incident only in the morning, presenting excellent development. Plant material collection began 6 months after planting the first seedlings. At the sixth month, the plants varied from 68 to 103 cm in height. At 8 months of age, the plant registered a maximum height of 180 cm. At each collection, 3 to 4 plants were cut at the height of 25 cm from the soil, and their leaves and thin branches (aerial part) were destined to the experiment predicted in this work. The regeneration of these plants took from 3 to 4 months.

### Essential oil yield vs climate parameters

**Fig. 1:**
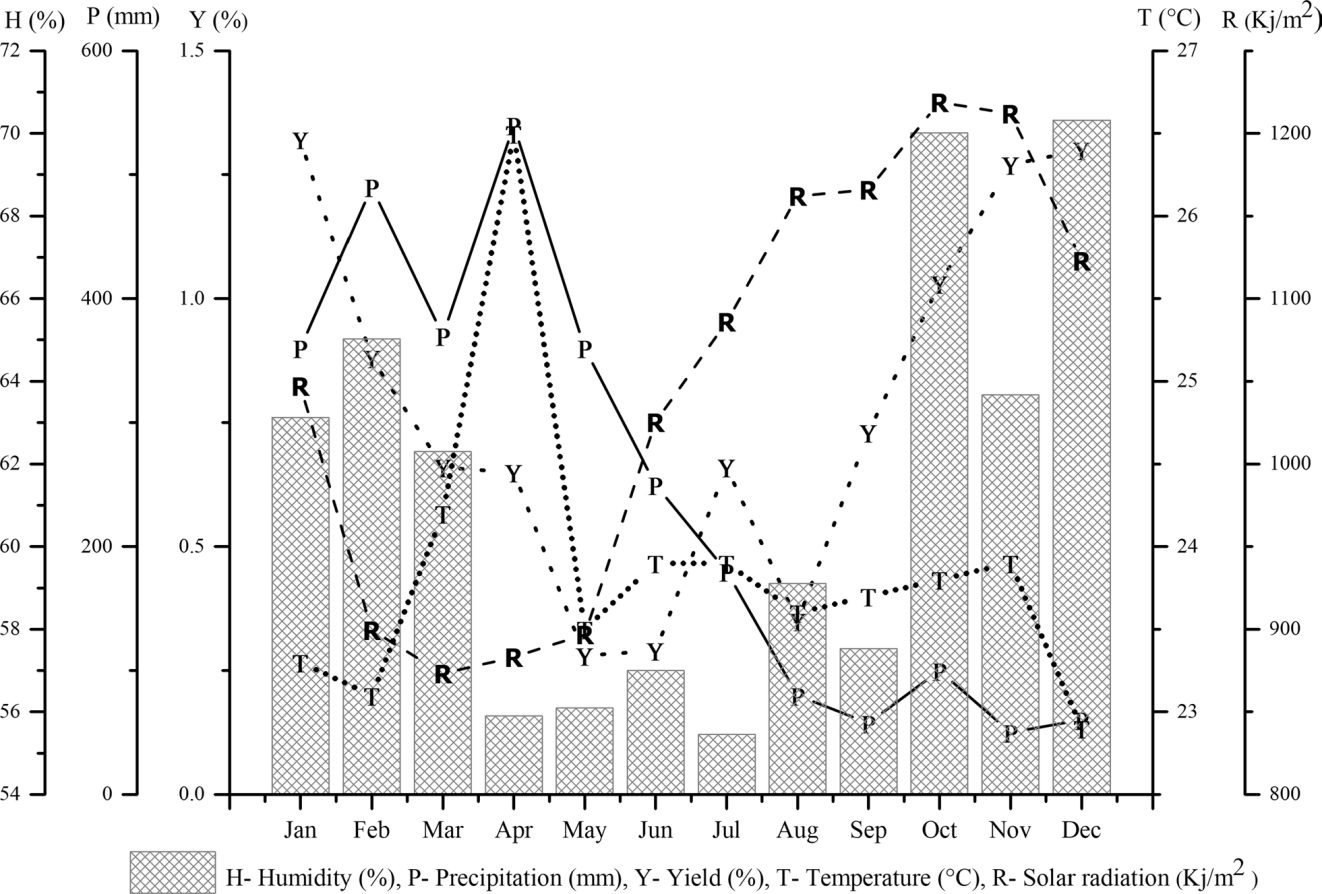
Essential oils yield (%) of *L. thymoides* and climatic variables measured at the time of collection: relative humidity (%); precipitation (mm); temperature (°C) and solar radiation (Kj/m^2^)

The climatic parameters, temperature, solar radiation, precipitation and relative humidity were monitored in the 12 months, to evaluate the seasonality in the yield and composition of *L. tymoides* essential oil. The mean values of temperature and solar radiation, between January and December, varied from 22.9 to 26.5 °C and 873.2 to 1123 kJ/m^2^, respectively. Likewise, mean relative humidity and mean rainfall ranged from 55.45 to 70.32% and 50 to 540 mm, respectively. Based on the precipitation data, the rainy season was from January to June, with a mean of between 250 and 540 mm, and the dry period was from July to December, varying between 50 and 180 mm. On the other hand, the temperature remained almost constant with an annual average of 23.86 °C ± 0.87 (Fig. 1).

In the Brazilian Amazon, only two seasons are considered throughout the year: a dry period and a rainy period and, among them, a few months of transition [19]. Due to the hot and humid climate of the region, precipitation is a parameter with high heterogeneity and significant variability of local and time. Thus, the dry period (called the Amazonian summer) and the rainy season (called the Amazonian winter) may present changes in its beginning and end.

The yield of the oils of *L. thymoides* in the seasonal study was 0.3% (May and June) to 1.3% (January, November, and December), with a mean of 0.7 ± 0.38% in the rainy season months, and 0.9 ± 0.36 in the months of the dry period (see Table 2). Thus, throughout the year, the yields of oils did not present a statistically significant difference between the two periods (p > 0.05). However, in the seasonal study, oil yield showed a strong correlation with the relative humidity (Table 1). In the circadian study, the oil yields were 0.7% (6 pm) to 1.1% (12 am) in the rainy season and from 0.5% (3 pm) to 0.9% (9 am) in the dry period. No statistical difference (p > 0.05) was observed in mean yields of the circadian study, which was 0.88 ± 0.13% in the rainy season and 0.72 ± 0.15% in the dry period. In the circadian survey, analyzing the yields of the oils about the collection times, a strong correlation directly proportional to the temperature (r^2^ 0.71) and a strong correlation inversely proportional to the humidity (r^2^ − 0.75) were observed, as can be seen in Table 1. Previously, studies with *L. thymoides* reported an oil yield of 0.71% for a sample collected in the city of Belém, State of Pará, Brazil (11) and an oil yield between 2.14 and 2.93% for another sample harvested in the city of Feira de Santana, State of Bahia, Brazil [10]. These differences in the yields of *L. thymoides* oils can be attributed to the diversity of the climatic factors in the plant collection areas.

**Table 1:** Correlation between climatic factors and seasonal and circadian studies, based on the yields of *L. thymoides* oils and thymol content (%)

Climatic factors	Seasonal study	Circadian study	Thymol
	Correlation coefficient (r^2^)		
Temperature (°C)	− 0.26	0.71	0.45
Precipitation (mm)	− 0.22		0.77*
Solar radiation (Kj/m^2^)	0.39		− 0.81*
Relative humidity (%)	0.74*	− 0.75	− 0.12

* Significant at *p* ≤ 0.05

### Composition of oils

The identification of the constituents of the oils by GC and GC–MS was on average 99.3% and 99.6% in the seasonal (S) and circadian (C) studies, respectively. In total, forty-five constituents were identified, and they are listed in Tables 2 and 3.

**Table 2:** Seasonal study of the *Lippia thymoides* oils during 12 months

Oil constituents (%)	Oil yields (%)													
	RI_C_	RI_L_	1.3	0.9	0.7	0.7	0.3	0.3	0.7	0.4	0.7	1.0	1.3	1.3
			Jan	Feb	Mar	Apr	May	Jun	Jul	Aug	Sep	Oct	Nov	Dec
(*3Z*)-Hexenol	853	850		0.1	0.1			0.1	0.1	0.1	0.1	0.1	0.1	0.1
α-Thujene	922	924	0.2	0.6					0.1	0.2	0.1	0.2	0.3	0.2
Sabinene	971	969		0.1								0.1	0.1	0.1
1-Octen-3-ol	979	974						0.1	0.1	0.1	0.1	0.1	0.1	0.1
Myrcene	987	988	0.5	1.0	0.2	0.3		0.1	0.3	0.8	0.6	0.6	0.8	0.7
α-Phellandrene	1000	1002		0.1					0.1	0.1		0.1	0.1	0.1
α-Terpinene	1015	1014	0.3		0.2	0.2	0.1	0.1	0.3	1.0	0.9	0.6	0.6	0.7
*p*-*Cymene*	*1022*	*1020*	*3.8*	*6.4*	*2.2*	*2.5*	*0.5*	*0.6*	*2.9*	*5.3*	*4.8*	*3.3*	*4.8*	*5.4*
Limonene	1025	1024				0.1	0.1	0.1		0.2	0.2	0.1	0.1	0.1
1,8-Cineole	1028	1026				0.4		0.5	0.6	0.7	0.5	0.4	0.2	0.2
(*E*)-β-Ocimene	1047	1044							0.1	0.1	0.1	0.1	0.1	0.1
*γ*-*Terpinene*	*1058*	*1054*	*3.8*	*4.9*	*1.8*	*2.0*	*0.5*	*0.5*	*3.2*	*5.9*	*6.4*	*4.1*	*4.5*	*5.0*
*cis*-Sabinene hydrate	1064	1065		0.1					0.1		0.1	0.2	0.1	0.2
Terpinolene	1087	1086						0.1	0.1	0.1	0.1	0.1	0.1	0.1
Linalool	1094	1095		0.1	0.1			0.1	0.1	0.1	0.1	0.1	0.1	0.1
Camphor	1143	1141						0.1	0.1	0.1	0.1	0.1	0.1	0.1
Borneol	1167	1165				0.1		0.1	0.1	0.1	0.1		0.1	0.1
Umbellulone	1169	1167	0.3	0.2	0.1		0.1	0.2			0.2	0.5		
Terpinen-4-ol	1176	1174	0.5	0.3	0.2	0.2	0.1	0.6	1.0	0.9	0.6	0.5	0.8	0.4
α-Terpineol	1186	1186						0.1	0.3		0.1	0.1	0.1	0.1
Thymol methyl ether	1231	1232	0.9	0.9	0.4	0.4	0.1	0.9	1.8	1.7	1.7	1.7	1.6	1.7
*Thymol*	*1296*	*1289*	*72.3*	*75.6*	*78.4*	*80.0*	*77.8*	*78.0*	*70.1*	*67.3*	*65.7*	*71.6*	*72.5*	*72.3*
*Thymol acetate*	*1354*	*1349*	*9.6*	*5.1*	*7.3*	*8.0*	*13.7*	*9.2*	*8.7*	*6.2*	*7.1*	*6.4*	*4.8*	*5.1*
Eugenol	1355	1356					0.2	0.1		0.1			0.1	0.1
α-Copaene	1372	1374	0.1				0.4	0.1	0.1	0.1	0.1	0.1	0.1	
Methyleugenol	1401	1403	0.1				0.1	0.1	0.1			0.1	0.1	0.1
*(E)*-*caryophyllene*	*1419*	*1417*	*4.5*	*2.9*	*6.2*	*4.1*	*4.5*	*4.1*	*5.5*	*5.4*	*5.5*	*4.9*	*4.4*	*3.9*
trans-α-Bergamotene	1433	1432	0.2		0.1		0.1	0.1	0.2	0.1		0.1	0.1	0.1
Aromadendrene	1439	1439						0.1	0.1	0.1	0.1	0.1	0.1	0.1
6,9-Guaiadiene	1442	1442	0.6											
α-Humulene	1454	1452		0.4	0.8	0.5	0.5	0.7	0.8	0.7	0.8	0.7	0.6	0.5
γ-Muurolene	1479	1478	0.1		0.1	0.1		0.1	0.2	0.2	0.2	0.1	0.1	0.1
Germacrene D	1485	1484	0.1	0.4	0.5	0.3	0.3	0.2	0.6	0.2	0.6	0.8	0.4	0.5
γ-Amorphene	1497	1495			0.1			0.1	0.1	0.1	0.1	0.1	0.1	0.1
Viridiflorene	1498	1496									0.1	0.1	0.1	0.1
α-Muurolene	1501	1500	0.1				0.1	0.1	0.1	0.1		0.1	0.1	0.1
δ-Amorphene	1512	1511						0.1	0.1	0.1	0.1	0.1	0.1	0.1
γ-Cadinene	1514	1513	0.1	0.1	0.1	0.1	0.1	0.1		0.1	0.2	0.1	0.1	0.1
δ-Cadinene	1524	1522	0.2	0.1	0.3	0.1	0.1	0.3	0.1	0.3	0.4	0.2	0.2	0.1
*trans*-Calamenene	1525	1521						0.1	0.1		0.1	0.1	0.1	
α-Cadinene	1538	1537						0.1	0.1	0.1	0.1	0.1	0.1	
Caryophyllene oxide	1582	1582	0.3			0.3	0.2	0.5	0.4	0.2	0.3	0.3	0.3	0.3
Humulene epoxide II	1607	1608		0.2	0.4		0.1	0.1	0.1	0.1	0.1	0.1	0.1	0.1
α-Cadinol	1654	1652	0.1		0.1			0.1	0.1	0.1	0.1	0.1	0.1	0.1
14-Hydroxy-9-*epi*-(*E*)-caryophyllene	1670	1668						0.1	0.1	0.1	0.1	0.1	0.1	0.1
Monoterpenes hydrocarbons			8.6	13.1	4.4	5.5	1.2	2.0	7.1	13.7	13.2	9.3	11.5	12.5
Oxygenated monoterpenes			83.6	82.3	86.5	88.7	91.8	89.3	82.9	77.1	76.3	81.6	80.4	80.3
Sesquiterpene hydrocarbons			6.0	3.9	8.2	5.2	6.1	6.3	8.1	7.6	8.4	7.7	6.7	5.8
Oxygenated sesquiterpenes			0.4	0.2	0.5	0.3	0.3	0.8	0.7	0.5	0.6	0.6	0.6	0.6
Other compounds			0.1	0.1	0.1		0.3	0.4	0.3	0.3	0.2	0.3	0.4	0.4
Total			98.7	99.6	99.7	99.7	99.7	98.8	99.1	99.2	98.7	99.5	99.6	99.6

RI_C_: calculated retention index (DB-5 ms column); RI_L_: literature retention index (Adams [17]); Main constituents in italics

**Table 3:** Circadian study of the *Lippia thymoides* oils on the rainy and dry seasons

Oil constituents (%)	Oil yields (%)													
	RI_C_	RI_L_	February—rainy season						September—dry season					
			0.9	0.9	1.1	0.9	0.7	0.8	0.7	0.9	0.8	0.5	0.8	0.6
			6 am	9 am	12 am	3 pm	6 pm	9 pm	6 am	9 am	12 am	3 pm	6 pm	9 pm
(*3Z*)-Hexenol	853	850	0.1	0.1	0.1	0.2	0.1	0.1	0.1		0.1			0.1
α-Thujene	922	924	0.6	1.0	1.2	1.4	0.7	1	0.1	0.2	0.5	0.7	0.4	0.8
α-Pinene	934	932		0.1	0.1	0.1		0.1		0.1	0.1	0.1	0.1	
Camphene	948	946			0.1	0.1					0.1		0.1	
Sabinene	971	969								0.1		0.1	0.1	
β-Pinene	978	974	0.1		0.2	0.2								0.1
1-Octen-3-ol	979	974							0.1	0.1	0.1	0.1	0.1	0.2
Myrcene	987	988	1.0	1.0	1.2	1.5	1.0	1	0.6	0.6	0.8	1.6	1.2	1.8
α-Phellandrene	1000	1002	0.1	0.1	0.1	0.1	0.1	0.1		0.1	0.1	0.1	0.1	0.1
*iso*-Sylvestrene	1007	1007							0.1	0.1	0.1	0.1	0.1	0.1
α-Terpinene	1015	1014		0.7		1.2	0.7	0.6	0.9	0.9	1.1	1.6	1.3	1.5
*p*-*Cymene*	*1022*	*1020*	*6.4*	*4.7*	*7.8*	*6.7*	*5.5*	*4.7*	*4.8*	*4.1*	*7.0*	*8.4*	*6.8*	*8.8*
Limonene	1025	1024				0.2			0.2	0.2	0.2	0.3	0.3	0.4
1,8-Cineole	1028	1026				0.3			0.5	0.3	0.4	0.6	0.6	0.8
(*E*)-β-Ocimene	1047	1044			0.1	0.1			0.1		0.1	0.1	0.1	0.1
*γ*-*Terpinene*	*1058*	*1054*	*4.9*	*4.9*	*5.8*	*6.8*	*4.8*	*4.9*	*6.4*	*7.0*	*8.3*	*8.4*	*8.0*	*7.6*
*cis*-Sabinene hidrate	1064	1063	0.1	0.1	0.1	0.1	0.1	0.1	0.1					
Terpinolene	1087	1086			0.1	0.1			0.1	0.1	0.1	0.1	0.1	0.1
Linalool	1094	1095	0.1	0.1	0.1	0.1	0.1	0.1	0.1	0.1	0.1	0.1	0.1	0.1
Camphor	1143	1141				0.1			0.1	0.1	0.1	0.1	0.1	0.1
Umbellulone	1169	1167	0.2	0.1	0.1	0.1	0.1	0.1	0.2	0.2	0.2		0.1	0.2
Terpinen-4-ol	1176	1174						0.1	0.1	0.1	0.1		0.1	0.3
α-Terpineol	1186	1186	0.4	0.1	0.2	0.3			0.6	0.5	0.4	0.6	0.4	
Thymol methyl ether	1231	1232	0.9	0.4	0.7	0.7	0.4	0.4	1.7	1.5	1.0	1.1	1.2	1.2
*Thymol*	*1296*	*1289*	*75.6*	*77.7*	*70.0*	*66.6*	*77.8*	*77.8*	*65.7*	*65.9*	*67.8*	*62.6*	*61.5*	*63.2*
*Thymol acetate*	*1354*	*1349*	*5.1*	*4.6*	*6.0*	*7.3*	*4.5*	*4.6*	*7.1*	*9.0*	*4.5*	*5.5*	*6.9*	*4.7*
Eugenol	1355	1354	0.1							0.1	0.1	0.1	0.1	
α-Copaene	1372	1374			0.1				0.1	0.1	0.1	0.1		
*(E)*-*Caryophyllene*	*1419*	*1417*	*2.9*	*3.3*	*3.8*	*3.6*	*3.0*	*3.3*	*5.7*	*5.1*	*4.1*	*4.3*	*6.3*	*4.8*
*trans*-α-Bergamotene	1433	1432			0.1	0.1				0.1	0.1	0.1	0.2	0.2
Aromadendrene	1439	1439							0.1	0.1	0.1			
α-Humulene	1454	1452	0.4	0.3	0.5	0.4	0.3	0.3	0.8	0.8	0.5	0.6	0.9	0.7
γ-Muurolene	1479	1478		0.1	0.1	0.1		0.1	0.2	0.1	0.1	0.1	0.2	0.1
Germacrene D	1485	1484	0.4	0.3	0.6	0.5	0.3	0.3	0.7	0.8	0.4	0.6	0.9	0.7
γ-Amorphene	1497	1495							0.1	0.1	0.1	0.1	0.1	
α-Muurolene	1501	1500			0.1					0.1	0.1	0.1	0.1	
δ-Amorphene	1512	1511							0.1		0.1	0.1		
γ-Cadinene	1514	1513	0.1		0.1	0.1			0.2	0.1	0.1	0.1	0.1	0.1
δ-Cadinene	1524	1522	0.1	0.1	0.1	0.1		0.1	0.4	0.2	0.2	0.2	0.3	0.2
α-Cadinene	1538	1537				0.1			0.1	0.1	0.1	0.1		
Humulene epoxide II	1607	1608							0.3	0.3	0.2	0.3	0.3	0.3
α-Cadinol	1654	1652	0.2	0.1	0.2	0.2	0.1	0.1	0.1	0.1	0.1	0.1	0.1	
14-Hydroxi-9-*epi*-(*E*)-caryophyllene	1670	1668						0.1	0.1	0.1	0.1	0.1	0.1	0.1
Monoterpene hydrocarbons			13.1	12.5	16.7	18.5	12.8	12.4	13.3	13.5	18.5	21.6	18.7	21.4
Oxygenated monoterpenes			82.4	83.1	77.2	75.6	83.0	83.2	76.2	77.7	74.6	70.6	71.0	70.6
Sesquiterpene hydrocarbons			3.9	4.1	5.5	5.0	3.6	4.1	8.5	7.7	6.1	6.5	9.1	6.8
Oxygenated sesquiterpenes			0.2	0.1	0.2	0.2	0.1	0.1	0.5	0.5	0.4	0.5	0.5	0.4
Other compounds			0.2	0.1	0.1	0.2	0.1	0.1	0.2	0.2	0.3	0.2	0.2	0.3
Total (%)			99.8	99.9	99.7	99.5	99.6	99.9	98.7	99.6	99.9	99.4	99.5	99.5

RI_C_: calculated retention index (DB-5 ms column); RI_L_: literature retention index (Adams [17]); Main constituents in italics

The predominance of oxygenated monoterpenes (S: 76.3–91.8%; C: 70.6–83.2%) was observed in the oils, followed by monoterpene hydrocarbons (S: 1.2–13.7%; C: 12.4–21.6%) and sesquiterpene hydrocarbons (S: 3.9–8.4%; C: 3.6–9.1%). Thymol (S: 65.7–80.0%; C: 61.5–77.8%), thymol acetate (S: 4.8–13.7%; C: 4.5–9.0%), γ-terpinene (S: 0.5–6.4%; C: 4.8–8.4%), *p*-cymene (S: 0.5–6.4%; C: 4.1–8.8%), and (*E*)-caryophyllene (S: 2.9–6.2%; C: 2.9–6.3%) were the principal compounds. The mean thymol content was higher in the rainy season (S: 77.0%; C: 74.3%) than in the dry period (S: 69.9%; C: 64.5%). The climatic variables that most influenced the thymol content were rainfall precipitation (directly proportional) and solar radiation (inversely proportional), as can be seen by the correlation data in Table 1.

A similar study with *Lippia origanoides* Kunth, collected in Santarém, State of Pará, Brazil, whose primary component was carvacrol, a thymol isomer, did not show a statistical difference for the two collection periods (rainy and dry seasons), regarding the carvacrol content [20]. Besides that, in previous works was observed that oils of *Lippia* species occurring in the Intercontinental Amazon have shown significant amounts of thymol, as *L. glandulosa* Schauer sampled in the Lavrado area of Roraima state, Brasil [21], *L. origanoides* Kunth (thymol-type) collected in Bucaramanga, Santander District, Colombia [22], and *L. gracilis* Schauer harvested in Balsas, Maranhão state, Brasil [23]. This way could consider that these thymol-type oils may result from the polymorphism of some different *Lippia* species, mainly taking into account the climatic factors of the collection sites.

### Variability in oil composition

**Fig. 2:**
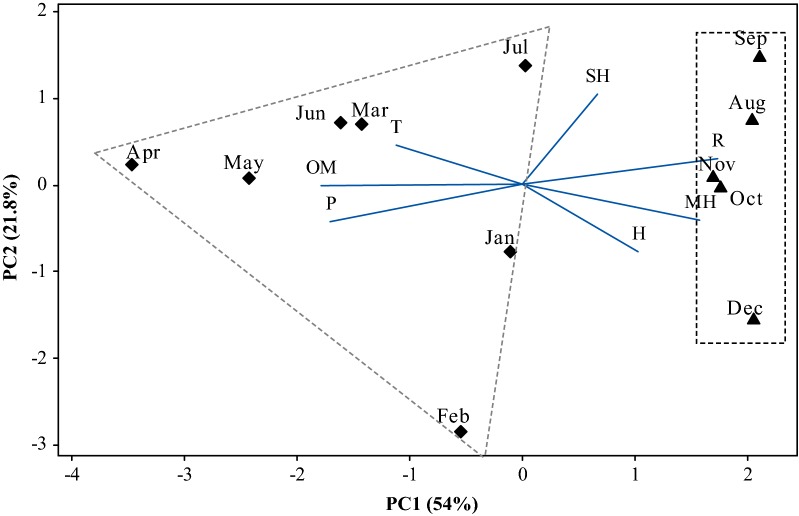
Biplot (PCA) resulting from the analysis of the classes of compounds identified in the oils of *L. thymoides* of the seasonal study, in association with temperature, solar radiation, relative humidity, and precipitation

**Fig. 3:**
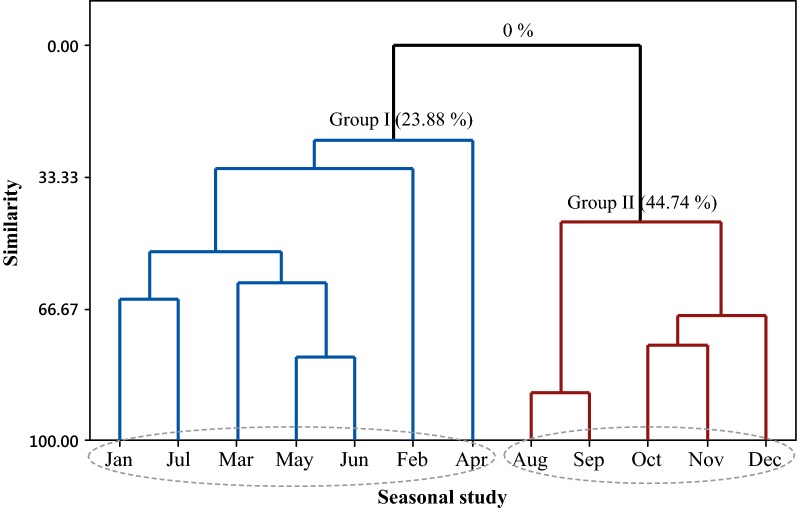
Dendrogram representing the similarity relationship of the classes of compounds identified in the oils of *L. thymoides* in the seasonal study, in association with temperature, solar radiation, relative humidity and precipitation

The multivariate analysis of PCA (Principal Component Analysis) (Fig. 2) and HCA (Hierarchical Cluster Analysis) (Fig. 3) was applied to the monoterpene hydrocarbons (MH), oxygenated monoterpenes (OM), and sesquiterpene hydrocarbons (SH), quantified in the oils of seasonal study, in association with temperature, solar radiation, relative humidity, and precipitation, the seasonal variables at the plant collection site. The main components (PC1 and PC2) presented a proportional variance of 54% and 21.8% respectively, and the total variation of 75.8% in the PCA analysis. The CP1 component was mainly responsible for separating the two groups formed in Fig. 2. The HCA analysis, considering the Euclidean distances and complete bonds, confirmed the formation of two distinct groups, as observed in the dendrogram of Fig. 3. Group 1 is associated with the variables from January to July, characterized by the higher content of oxygenated monoterpenes (82.3–91.8%) and is related to temperature variation and precipitation. During these months, a low temperature was registered, between 23.1 and 26.5 °C, and the highest level of precipitation, between 180 and 540 mm. The group II, represented by the months of August to December, is characterized by the higher content of monoterpene hydrocarbons (9.3 to 13.7%) and sesquiterpene hydrocarbons (5.8 to 8.4%), and related to a low relative humidity (57.5 to 70.3%) and to the highest solar radiation (1123 to 1219 kJ/m^2^) observed in the seasonal period.

The composition of the oils in the circadian study, during the rainy (R) and dry (D) periods (Table 3), presented on average the following primary constituents: thymol (R: 74.3%; D: 64.5%), γ-terpinene (R: 5.4%; D: 7.6%), thymol acetate (R: 5.4%; D: 6.3%), *p*-cymene (R: 6.0%; D: 6.7%), and (*E*)-caryophyllene (R: 3.3%; D: 5.1%). Thymol showed a higher percentage in the rainy season, while γ-terpinene, thymol acetate, *p*-cymene and (*E*)-caryophyllene showed higher levels in the dry period.

**Fig. 4:**
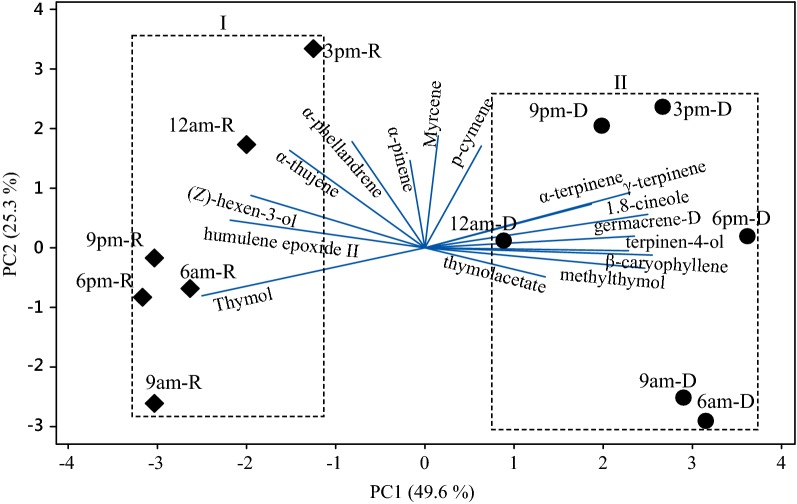
Biplot (PCA) resulting from the analysis of the oil constituents of *L. thymoides* in the circadian study, during the rainy (R, February) and dry (D, September) seasons

**Fig. 5:**
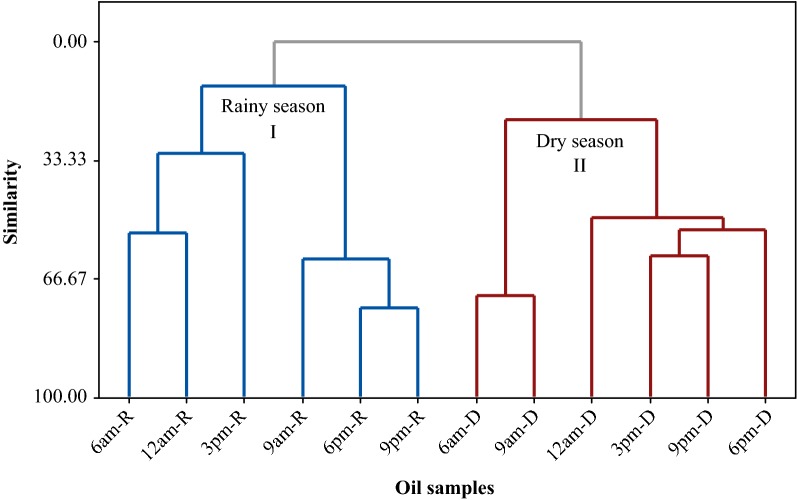
Dendrogram representing the similarity relationship of the oils composition of *L. thymoides* in the circadian study, during the rainy (R, February) and dry (D, September) season

Similarly, PCA and HCA studies were applied to the constituents identified in oils from the rainy and dry periods of the circadian study (Figs. 4 and 5). The main components (PC1 and PC2) presented a proportional variance of 49.6% and 25.3% respectively, and the total variation of 74.9% in the PCA analysis. The HCA analysis, considering the Euclidean distances and complete bonds, confirmed the formation of two distinct groups, as observed in the dendrogram of Fig. 5. Group I was formed with the constituents of the oils resulting from *L. thymoides* collections, in a daily cycle of the rainy season (February), characterized by the higher thymol content, followed by the minor percent of (*Z*)-hexen-3-ol, α-thujene, α-pinene, α-phellandrene and humulene epoxide II. Group II resulted from the grouping of the oils of the plant samples collected during 1 day in the dry period (September) and it was characterized by a higher content of *p*-cymene, γ-terpinene, thymol acetate and (*E*)-caryophyllene, followed by a lower percentage of myrcene, α-terpinene, 1,8-cineole, terpinen-4-ol, methylthymol, and germacrene D.

It is widely known that essential oils can vary in composition depending on the place, time of day and seasonality. Therefore, these different chemical profiles must be associated with the environmental conditions existing at their respective collection sites. The knowledge of this variation in the composition of *L. Thymoides* oil is essential from the ecological and taxonomic point of view, regarding the management and economic use of the species.

As already mentioned, *Lippia thymoides* is a species little studied from the phytochemical point of view. Literature report methylthymol as the main constituent of the essential oil of a specimen of *L. thymoides* described to Brazil, but with an unknown collection site [24, 25]. Two other specimens with occurrence in the State of Bahia, Brazil, were reported to have essential oil rich in (*E*)-caryophyllene [9, 10]. Also, there is another citation of a specimen collected in the State of Pará, Brazil, whose main constituent was thymol [11]. Thus, based on the information obtained in the literature and the present study, the essential oil of *L. thymoides* has shown the following chemical types: methylthymol, (*E*)-caryophyllene and thymol.

Thymol, methylthymol, thymol acetate, *p*-cymene, and γ-terpinene are all monoterpene constituents that occur together in many other essential oils, particularly in *Lippia* species [21–23]. All these constituents are derived from the same biosynthetic pathway in the plant, where γ-terpinene is considered the biogenetic precursor of the other monoterpenes [26, 27].

## Conclusions

Planting of *L. thymoides* showed excellent development, reaching about 1 m in length in 6 months. On average, the oil yield was 0.7% in the rainy season and 0.9% in the dry period, showing no significant statistical difference. In the seasonal study, the oil yield presented a strong correlation with the relative humidity. In the circadian evaluation, the correlation was with the temperature. In the annual survey, the rainy period showed the highest content of oxygenated monoterpenes, in association with the temperature and precipitation of the planting local. The mean thymol content was higher in the rainy season than in the dry period. The climatic variables that most influenced the thymol content were rainfall precipitation and solar radiation. These different chemical profiles must be associated with the environmental conditions existing at their respective collection sites. The knowledge of this variation in the composition of *L. Thymoides* oil is essential from the ecological and taxonomic point of view, regarding the management and economic use of the species.

## Abbreviations

HCA: Hierarchical Cluster Analysis
PCA: Principal Component Analysis
GC: Gas chromatography
GC–MS: Gas chromatography–Mass spectrometry
INMET: Instituto Nacional de Meteorologia
EIMS: eletron ionization mass spectrometry
R: rainy season
D: dry season

## References

[1] O’Leary N, Denham SS, Salimena F, Múlgura ME (2012). Species delimitation in *Lippia* section *Goniostachyum* (Verbenaceae) using the phylogenetic species concept. Bot J Linn Soc.

[2] http://floradobrasil.jbrj.gov.br/reflora. Accessed July 2018

[3] Funch LS, Harley RM, Funch R, Giulietti AM, Melo E (2004). Plantas Úteis da Chapada Diamantina.

[4] Salimena FRG, Mulgura M. *Lippia*. In: Lista de Espécies da Flora do Brasil. Jardim Botânico do Rio de Janeiro. 2015. http://floradobrasil.jbrj.gov.br/jabot/floradobrasil/FB21457. Accessed 11 Apr 2018

[5] de Almeida MZ (2011). Plantas Medicinais.

[6] Costa PS, Souza EB, Brito EHS, Fontenelle ROS (2017). Atividade antimicrobiana e potencial terapêutico do gênero *Lippia* sensu lato (Verbenaceae). Hoehnea.

[7] Gobbo-Neto L, Lopes NP (2007). Plantas medicinais: fatores de influência no conteúdo de metabólitos secundários. Quím Nova.

[8] Hussain AI, Anwar F, Hussain Sherazi ST, Przybylski R (2008). Chemical composition, antioxidant and antimicrobial activities of basil (*Ocimum basilicum*) essential oils depends on seasonal variations. Food Chem.

[9] Craveiro AA, Fernandes AG, Andrade CHS, Matos FJA, Alencar JW, Machado MIL (1981). Óleos essenciais de Plantas do Nordeste.

[10] Silva FS, Menezes PMN, de Sá PGS, Oliveira ALDS, Souza EAA, da Silva JRGA, de Lima JT, Uetanabaro APT, Silva TRDS, Peralta ED, Lucchese AM (2015). Chemical composition and pharmacological properties of the essential oils obtained seasonally from *Lippia thymoides*. Pharm Biol.

[11] Zoghbi MGB, Andrade EHA, Zoghbi MGB, Mota MGC, Conceição CCC (2014). Composição química dos óleos essenciais de plantas aromáticas comercializadas no Ver-o-Peso. Plantas aromáticas do Ver-o-Peso.

[12] Menezes PMN, Oliveira HR, Brito MC, Paiva GO, Ribeiro LAA, Lucchese AM, Silva FS (2018). Spasmolytic and antidiarrheal activities of *Lippia thymoides* (Verbenaceae) essential oil. Nat Prod Res.

[13] Pinto CDP, Rodrigues VD, Pinto FDP, Pinto RDP, Uetanabaro APT, Pinheiro CSR, Gadea SFM, Silva TRDS, Lucchese AM (2013). Antimicrobial activity of *Lippia* species from the Brazilian semiarid region traditionally used as antiseptic and anti-infective agents. J Evid Based Complement Altern Med.

[14] Silva FS, de Menezes PMN, Sá PGS, Oliveira ALDS, Souza EAA, Bamberg VM, de Oliveira HR, de Oliveira SA, Araújo REE, Uetanabaro APT, da Silva TR, Almeida JRGDS, Lucchese AM (2015). Pharmacological basis for traditional use of the *Lippia thymoides*. J Evid Based Complement Altern Med.

[15] Biasi LA, Costa G (2003). Propagação vegetativa de *Lippia alba*. Cienc Rural.

[16] Maia JGS, Andrade EHA (2009). Data base of the Amazon aromatic plants and their essential oils. Quím Nova.

[17] Adams RP (2007). Identification of essential oil components by gas chromatography/mass spectrometry.

[18] NIST—National Institute of Standars and Technology (2011). Mass Spectral Library (NIST/EPA/NIH, v.2.0d).

[19] Loureiro RS, Saraiva JM, Saraiva I, Senna RC, Fredó AS (2014). Estudo dos eventos extremos de precipitação ocorridos em 2009 no Estado do Pará. Rev Bras Meteorol.

[20] Sarrazin SLF, da Silva LA, de Assunção APF, Oliveira RB, Calao VYP, da Silva R, Stashenko EE, Maia JGS, Mourão RHV (2015). Antimicrobial and seasonal evaluation of the carvacrol-chemotype oil from *Lippia origanoides* Kunth. Molecules.

[21] Maia JGS, da Silva MHL, Andrade EHA, Carreira LMM (2005). Essential oil variation in *Lippia glandulosa* Schauer. J Essent Oil Res.

[22] Stashenko EE, Martínez JR, Ruíz CA, Arias G, Durán C, Salgar W, Cala M (2010). *Lippia origanoides* chemotype differentiation based on essential oil GC-MS and principal component analysis. J Sep Sci.

[23] Franco CS, Ribeiro AF, Carvalho NCC, Monteiro OS, da Silva JKR, Andrade EHA, Maia JGS (2014). Composition and antioxidant and antifungal activities of the essential oil from *Lippia gracilis* Schauer. Afr J Biotechnol.

[24] Terblanché FC, Kornelius G (1996). Essential oil constituents of the genus *Lippia* (Verbenaceae)—a literature review. J Essent Oil Res.

[25] Pascual ME, Slowing K, Carretero E, Sánchez Mata D, Villar A (2001). *Lippia*: traditional uses, chemistry and pharmacology—a review. J Etnopharmacol.

[26] Poulose AJ, Croteau R (1978). Biosynthesis of aromatic monoterpenes. Arch Biochem Biophys.

[27] Poulose AJ, Croteau R (1978). γ-Terpinene synthetase: a key enzyme in the biosynthesis of aromatic monoterpenes. Arch Biochem Biophys.

